# Collectanea Medica

**Published:** 1814-01

**Authors:** 


### COLLECTANEA MEDICA,

CONSISTING OF

### ANECDOTES, FACTS, EXTRACTS, ILLUSTRATIONS, QUERIES, SUGGESTIONS, &c.

RELATING TO THE

*History or the Art of Medicine, and the Auxiliary Sciences.*

### *Case of extraordinary Abstinence.*

CICELY DE RYDGEWAY, in the 31st Edward III. being indicted and condemned for killing her husband, fasted forty days together in Arcta Prisona, without any

 meat meat or drink. Of this extraordinary abstinence, the copy of the following Record, lodged in the Tower of London, is a proof.

Ex Rot. de Anno Regni Regis Edwardi Tertii 31 Partet, Memb. 11.

Rex omnibus ballivis et fidelibus suis, ad quos, &c. Salutem sciatis quod cum Cecilia quæ fuit uxor Johannis de Rygeway, nuper indictata de morte ipsius Johannis viri sui, et de morte illa coram dilecti et fidelibus nostris Henrico Grove, et sociis suis justic. nostris gaolam nostram Notyngh. deliberand. assign. allocuta, pro eo quod se tenuit mutam ad pænam suam extitit adjudicata, ut dicitur, in qua sine cibo et potu, in Arcta Prisona per quadraginta dies vitam sustinuit, via miraculi, et quasi contra naturam humanam, sicut ex testimonio accepimus fide digno. Nos ea de causa, pietate moti ad laudem Dei, et gloriosæ Virginis Mariæ matris suæ, unde dictum miraculum processit ut creditur. De gratia nostra speciali pardonavimus eidem Ceciliæ executionem judicis prædicti; volentes quod eadem Cecilia a prisona prædicta deliberetur, et de corpore pro ulterius non sit impetita, occasione judicii supradicti in cujus, &c. Testimonium R. apud West. xxv die Aprilis.

*Per. Brc. de Privato Sigillo.*

*Convenit cum Record.*

LAUR. HALSTEAD, Deput.

ALGERN. MAY, Mil.

---

*Diseases of the Laplanders.*

It is curious to know the diseases to which a people are liable, that lead a life so different from ours, and that are strangers to all the baneful effects of luxury and vice, so prevalent among the civilised nations of Europe. Fevers are very rare, and so is the small-pox; which, from the great distance at which these people live from each other, cannot make its way through the community as it does in towns and populous districts. Hence, there are many Laplanders who have never had that disease, and who are liable to fall a sacrifice to it, when exposed to its infection. Intermittent fevers are rare; the coldness of the country counteracting the baneful effects of marshes and stagnant waters, in which Lapland abounds.

Swelled necks, similar to those that occur in Switzerland, and which are there known by the name of *goitres*, are frequent. They have been ascribed to the use of snow-water, but this must be a vulgar prejudice. It is much more likely that they are produced by something peculiar in the dress of cold countries; since it is in them only that they abound.

Sore or *blear* eyes is a disorder almost universal among the Laplanders, and is to be ascribed to two causes, either of which would probably be sufficient to produce a disorder in the eyes. Their huts or tents are constantly filled with a thick cloud of wood smoke. Now every person that has ever been subjected to the influence of this smoke, knows that it is peculiarly acrid, abounding in the fumes of vinegar, and of an empyreumatic oil; both of which act upon the eyes with great energy, and occasion a constant smarting. This I conceive to occasion the blear-eyed appearance which distinguishes almost all the Laplanders. During seven months of the year, the ground is covered with snow; the glittering of which, and the great quantity of light which it reflects, has a pernicious effect upon the eyes, weakens them, and is the probable reason why many of the Laplanders become blind in their old age. The absence of the sun during a part of the winter no doubt diminishes this evil, though it does not remove it entirely.

Female obstructions are rare, though sometimes met with among the better sort of people. It is said that the *catamenia* are much more scanty in Lapland than in the southern parts of Europe. Female diseases from debility are unknown. There are even instances of women walking twenty-five miles to church within five days after being delivered of a child.

Hysterics sometimes occur, but very rarely. Epilepsy is more common. Head-aches are frequent; and deafness is a common disease of old people. A swelling or falling down of the uvula is not uncommon. They cure it by cutting off the part affected.

Coughs and colds are exceedingly uncommon, notwithstanding the coldness of the climate, and the small means which they have of guarding against it. Cases of consumption now and then occur, but by no means so frequently as in Great Britain. It would be of importance to know whether a greater proportion of children die in that country than here; because it would enable us to ascertain whether the greater proportion of health which the Laplanders enjoy be owing to those of weakly constitutions dying in their infancy, or to their really having in general a stronger constitution than those nations among whom luxury and vice have made greater progress. I have little doubt that the first is the true cause, as it is of the great health which savages in general enjoy.

Pleurisies are common in spring and autumn; and lumbago frequently makes its appearance in summer. Asthma is not uncommon among old people; and cases of hoarseness now and and then occur in winter and spring. Disorders of the stomach and bowels are not unfrequent; but they seldom last long. The stone, the gout, the jaundice, are entirely unknown to them. The absence of the stone deserves the attention of medical men. Some have ascribed this disease to the use of malt or fermented liquors; an opinion so far confirmed by its never appearing in Lapland, where fermented liquors are unknown. At the same time, reflexion will convince us that little stress can be laid upon such reasoning. Our knowledge of the Lapland diseases is necessarily very limited, being merely the information picked up by three or four medical men, during a journey through the country. The stone might easily have existed in Lapland without coming under their observation. The natives may be afflicted with the disease without ascribing it to the true cause. The existence of the stone in the inferior animals, even in those that go wild, and whose food, therefore, has not been altered by the interference of man, ought to prevent us from hastily ascribing the disease to any peculiar species of food. It is found both in graminivorous and carnivorous animals; though much more frequently, I believe, in the former than in the latter. We cannot, therefore, ascribe its absence among the Laplanders to their living on animal food almost entirely; though that may contribute somewhat, perhaps.

The great remedy which the Laplanders employ in most of their diseases is the actual cautery; a remedy much used by the ancient physicians, and still famous in Japan, where the substance employed for the purpose is called *moxa*. This substance, known in Lapland by the name of *toule*, is made of a fine fungus found on the birch, and always chosen from the south side of the tree. Of this they apply a piece as large as a pea upon the afflicted part, setting fire to it with a twig of birch, and letting it burn gradually away. This is repeated two or three times. It produces a sore that will often keep open for six months; nor must it be closed till it heals spontaneously. This remedy is used for all aches and pains, the head-ache, tooth-ache, pleurisy, pain in the stomach, lumbago, &c.

Sometimes they apply a kind of plaster called *kattie*, which is made in the following manner. The fine loose scaly bark of the birch is set on fire, and immediately quenched in water. It is then chewed, and afterwards mixed with fresh turpentine from the spruce fir, both being kneaded together by the hands, till the mass becomes a black uniform plaster. This is successfully applied to hard imposthumes, &c. which it brings to maturity without pain, in a short time, and promotes their discharge.

*Tables*

*Tables of the mean Temperature of each Month of the Year in Stockholm and Petersburgh, cities situated nearly in the same latitude, and nearly at the same distance from the sea.*— Both tables are taken from the registers kept in the two capitals, by the Academies of Sciences, who hold their meetings in the respective cities. The Stockholm register is the mean of fifty years, the St. Petersburgh of twenty years; so that both are for a period sufficiently long to give very nearly the correct mean.

| | Mean height of Fahrenheit's thermometer at Petersburgh. | Stockholm. |
|---|---|---|
| January | 12·1° | 24° |
| February | 16·1 | 26·5 |
| March | 22 | 29·5 |
| April | 35.5 | 38·5 |
| May | 43·5 | 52·5 |
| June | 54 | 59·5 |
| July | 64·6 | 64 |
| August | 61·5 | 58·5 |
| September | 51 | 50·7 |
| October | 39·4 | 41·5 |
| November | 27·4 | 31·5 |
| December | 18·4 | 26·5 |
| Annual mean | 37·1 | 41·93 |

*Rain in Sweden.*

From the flatness of the country, the smallness of the rivers, and the apparent diminution of the lakes, there is reason to believe that the quantity of rain which falls annually in Sweden is not great. This agrees with the registers which I have seen. The following little table exhibits the depth of rain in English inches, which falls annually in the southern, middle, and northern districts of Sweden:

| | Rain. |
|---|---|
| Lund | 18·29 inches. |
| Upsala | 15·37 |
| Uleaborg | 14·35 |

*Thomson's Travels in Sweden.*

CRITICAL

